# DataAtlas: automatic generation of data dictionaries using large language models

**DOI:** 10.1093/jamiaopen/ooag119

**Published:** 2026-06-27

**Authors:** Raffaele Giancotti, Rajna Fani, Rafi Al Attrach, Pierangelo Veltri, Tom Pollard

**Affiliations:** Laboratory for Computational Physiology, MIT Institute for Medical Engineering and Science, Massachusetts Institute of Technology, Cambridge, MA 02139, United States; Department of Computer Engineering, Modeling, Electronics, and Systems Engineering, Università della Calabria, Arcavata di Rende (CS), 87036, Italy; Laboratory for Computational Physiology, MIT Institute for Medical Engineering and Science, Massachusetts Institute of Technology, Cambridge, MA 02139, United States; TUM School of Computation, Information and Technology, Technical University of Munich, Munich, Bavaria 80333, Germany; Laboratory for Computational Physiology, MIT Institute for Medical Engineering and Science, Massachusetts Institute of Technology, Cambridge, MA 02139, United States; TUM School of Computation, Information and Technology, Technical University of Munich, Munich, Bavaria 80333, Germany; Department of Computer Engineering, Modeling, Electronics, and Systems Engineering, Università della Calabria, Arcavata di Rende (CS), 87036, Italy; Laboratory for Computational Physiology, MIT Institute for Medical Engineering and Science, Massachusetts Institute of Technology, Cambridge, MA 02139, United States

**Keywords:** data dictionary, large language models, generative AI, clinical data

## Abstract

**Objectives:**

Reuse of shared datasets is often limited by incomplete or inconsistent documentation, hindering interpretation and secondary analysis. We developed DataAtlas, an open-source system for automated generation of data dictionaries from tabular datasets.

**Materials and Methods:**

DataAtlas combines deterministic structural profiling with large language model (LLM)-based semantic inference to generate human-readable descriptions of datasets, tables, and variables. The system integrates column metadata, statistical summaries, and representative sample values to produce concise, context-aware descriptions. We evaluated the approach on 3 heterogeneous clinical datasets using structural validation, blinded LLM-based comparison with official documentation, human expert review, and downstream task evaluation.

**Results:**

Generated descriptions were frequently preferred over official documentation, particularly when existing descriptions were incomplete or ambiguous. Human expert review showed strong agreement with high-performing models, with most descriptions rated as correct and low rates of hallucination. The system accurately reconstructed schema elements and flagged inconsistencies in existing documentation. In a text-to-SQL benchmark, augmenting database schemas with generated data dictionaries improved execution accuracy from 0.52 to 0.88.

**Discussion:**

Automated data dictionary generation improved both dataset interpretability and downstream analytical performances. Results highlight the importance of column-level metadata, particularly representative sample values, in grounding LLM-generated descriptions. Limitations include sensitivity to sample quality, potential domain-specific misinterpretation, and variability across model architectures, indicating that generated outputs should augment rather than replace expert curation.

**Conclusion:**

DataAtlas provides a practical approach for generating structured data dictionaries, enhancing the accessibility, reproducibility, and reuse of clinical data.

## Introduction

While the reuse of shared datasets is central to modern scientific research, the practical value of data sharing depends not only on access to data files but also on clear and structured documentation. In many repositories, datasets remain difficult to interpret because metadata are sparse, inconsistent, or missing. In a recent survey, more than 13% of datasets had no description and roughly 10% had only a very short one.^1^ As a result, substantial analytic effort is often spent on understanding and preparing data rather than on modeling or interpretation.^2^ Poor documentation therefore limits reproducibility, hinders collaboration, and reduces the impact of data-sharing.

Data dictionaries provide a practical solution. They describe the meaning, data types, constraints, coding schemes, and relationships of variables within a dataset.^3–5^ This supports reproducible research, facilitates secondary analyses, and improves opportunities for discovery and reuse. Their value is especially evident in data repositories, where researchers must navigate large collections of datasets, and in time-constrained settings such as datathons, where participants must rapidly assess unfamiliar data before analysis.^6^ Despite their value, comprehensive data dictionaries are often unavailable because their creation remains largely manual and time-consuming.^7^ Researchers are therefore frequently confronted with raw CSV files, incomplete field names, and outdated documentation, leaving a gap between shared tabular data and the structured representations needed for effective reuse.

Recent advances in large language models (LLMs), such as GPT,^8^ Gemini,^9^ and open-source models such as Llama, have demonstrated impressive capabilities in parsing and generating natural language, making them suitable for interpreting unstructured or semi-structured data.^10^^,^^11^ In clinical applications, LLMs have been used for tasks such as clinical note summarization, terminology mapping, and code prediction.^12–14^ However, their potential in automating metadata generation and inferring semantic relationships in tabular datasets remains underexplored. LLMs can disambiguate column names, interpret abbreviations, and generate consistent human-readable descriptions beyond the capabilities of rule-based approaches.

In this article, we introduce DataAtlas, an automated system for generating structured data dictionaries from collections of tabular files. Given a set of tables, DataAtlas: generates human-readable documentation at the dataset, table, and column levels; infers likely meanings, data types, and abbreviations; and identifies semantic relationships across tables using shared keys, contextual cues, and similarity signals. The resulting documentation can be exported in both machine-readable formats (eg, JSON) and human-readable formats (eg, Markdown), together with a visual representation of inferred relational structure.

By automating the creation of data dictionaries, DataAtlas aims to reduce the manual effort required to understand and document complex datasets, helping to maximize their accessibility and potential for reuse.

## Methods

**Figure 1.**
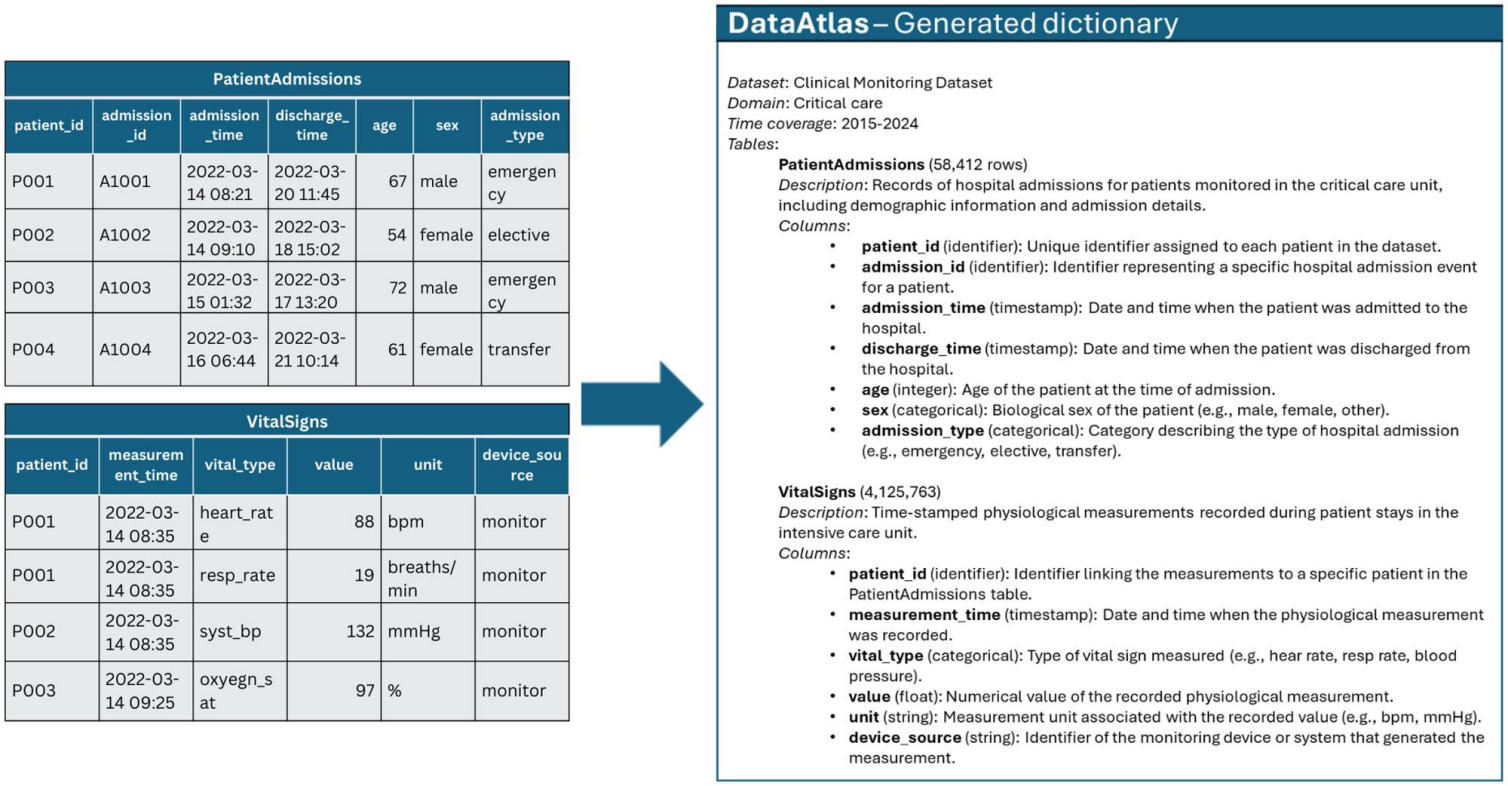
DataAtlas generates a comprehensive data dictionary from an input dataset. The dictionary can be exported in multiple formats, including human-readable markdown (shown here).

DataAtlas provides an automated pipeline for generating structured data dictionaries from tabular datasets. The system combines deterministic structural analysis, statistical profiling, and LLM-based semantic inference to produce human-readable descriptions of datasets, tables, and variables (Figure 1 shows an example of the output).

The pipeline comprises 3 key stages: (1) automated metadata extraction from raw tabular data, (2) LLM-based semantic description generation, and (3) structural and semantic evaluation of the resulting metadata.

### System architecture

#### Dataset ingestion and structural profiling

DataAtlas accepts tabular data in CSV and Excel. Since it assumes a relational tabular data model, JSON inputs are accepted only when they represent flat tabular data; nested objects and arrays are not supported and must be pre-flattened. Each file is treated as a table and processed through a multi-stage extraction pipeline designed to capture structural and statistical metadata. First, a hierarchical inference engine classifies column values into primitive types (integer, float, boolean, datetime, or string) by evaluating string patterns and removing formatting noise. Second, the system extracts structural patterns from column values. Each column value is converted into a normalized pattern mask, mapping digits to **9**, uppercase letters to **A**, lowercase letters to **a**, and retaining other characters (eg, “5257/1/2” → “9999/9/9”). Column-wise pattern frequency distributions are then analyzed to identify structured identifiers, clinical codes, timestamps, and other semantically meaningful data types. Finally, a heuristic classifier combines column-name keywords (eg, mrn, bp, age) with structural pattern matching to assign semantic tags such as identifier, clinical_measurement, icd_code, or timestamp.

#### Description generation

After structural metadata is extracted, DataAtlas generates natural-language descriptions for datasets, tables, and columns. The description generation module integrates schema metadata, statistical summaries, and representative sample values into structured prompt templates.

DataAtlas operates exclusively on information derived from the input dataset. Each prompt includes contextual information such as dataset name, table structure, column name, inferred data type, and example values. Statistical information (eg, null percentages, value ranges, or common categorical values) is also provided to help the model infer semantic context and measurement units. No external knowledge bases, reference databases, or additional data sources are accessed during generation.

To ensure concise and consistent dictionary entries, model outputs are restricted to a single descriptive sentence and post-processed to remove extraneous tokens. Multiple model architectures can be used through a unified dispatcher interface, including proprietary APIs (eg, *GPT-4*, *GPT-4o-mini*), cloud models (eg, *Gemini-2.5-Flash*), and locally hosted models (eg, *Llama 3.1* executed via Ollama). Table 1 illustrates a representative output generated by DataAtlas.

**Table 1.** Example metadata extraction for the CarpeDiem clinical dataset.

Column name	Data type	Semantic type	Generated description	Sample values
**Patient/ICU stay/ICU day**	Datetime	Timestamp	Timestamp marking specific patients ICU stay and day within that stay.	5257/1/2, 7705/2/3
**Patient_id**	Integer	Identifier	Unique identifier for each patient in the healthcare dataset.	8448, 9441, 262
**Age**	Integer	Age	Patient age in years, ranging from young adults to elderly individuals.	64, 49, 54
**COVID_status**	Float	Identifier	Binary identifier for patients COVID-19 status (1.0 positive, 0.0 negative).	0.0, 1.0

DataAtlas identifies structural and semantic types and generates concise, context-aware descriptions through its LLM-based generation module.

#### Metadata reporting

For each column, the pipeline generates a statistical profile, including central tendency and dispersion for numeric data and frequency-based summaries for categorical data. In addition, the pipeline reports missing data patterns by computing the null percentage for each column and identifies potential outliers in numeric variables using the interquartile range (IQR) method. DataAtlas also supports LLM-based discovery of relationships across tables (see Appendix B).

#### Implementation and reproducibility

DataAtlas is implemented in Python 3.11. Data manipulation and profiling operations are performed using pandas, while metadata schemas and structural constraints are enforced through pydantic, enabling formal validation of extracted fields and type consistency.

The architecture is hardware-agnostic. For the experiments reported in this study, local inference was performed using *Ollama* on a system equipped with an NVIDIA GeForce GTX 1650 Ti GPU (4 GB VRAM). This configuration demonstrates that the pipeline can be executed on consumer-grade hardware without requiring specialized high-performance computing infrastructure.

The generated data dictionary is exported both as a machine-readable JSON file and as a human-readable markdown report, ensuring compatibility with downstream automated workflows as well as manual clinical review. The complete source code and documentation required to reproduce the experiments are publicly available at https://github.com/rafgia/data-dictionary-generator.

### Evaluation framework

Evaluating automated data dictionary generation presents challenges due to the absence of standardized benchmarks and the inherently interpretative nature of natural language descriptions. To address this, we designed a 3-tiered evaluation framework assessing: (1) structural reconstruction accuracy, (2) semantic correctness and hallucination risk, and (3) comparative performance across expert-curated documentation and differing LLM architectures.

#### Model selection and benchmarking

To evaluate robustness and flexibility, we benchmarked 4 diverse LLM architectures: *GPT-4* and *GPT-4o-mini* (OpenAI), *Gemini-2.5-Flash* (Google), and *Llama 3.1 8B* (Meta, running locally via Ollama). This set covers a spectrum of computational requirements, from state-of-the-art proprietary models to efficient open-source local alternatives, enabling assessment of how model size and quantization impact the accuracy and conciseness of generated clinical descriptions.

#### Comparison with expert-curated documentation

To assess structural reliability across heterogeneous clinical domains, we evaluated the system on 3 datasets obtained from PhysioNet.^15^ The selected datasets, described in section “Evaluation datasets,” include expert-curated data dictionaries that served as a reference for benchmarking schema reconstruction. The restricted-access nature of these resources reduces the likelihood of overlap with LLM training corpora, thereby reducing potential data leakage effects. Structural evaluation focused on the accurate reconstruction of database schemas, data types, and primary key constraints directly from raw tabular files.

#### Evaluation datasets

We evaluated DataAtlas across 3 heterogeneous clinical datasets to assess robustness across distinct data structures and medical domains: ORCHID (n=64 columns), CarpeDiem (n=101 columns), and INSPIRE (n=62 columns). These datasets differ in size, temporal granularity, and relational complexity, providing a broad testbed for evaluating semantic description generation and relational inference.

- ORCHID (Organ Retrieval and Collection of Health Information for Donation)^16^ is a clinical database focused on the organ procurement process in the United States. It contains case-level data from 6 Organ Procurement Organizations (OPOs) between 2015 and 2021, including demographics; clinical timestamps (referral, evaluation, consent, procurement, and transplant), laboratory measurements; event-level clinical variables; and regional mortality estimates. The dataset supports research on efficiency, equity, and decision-making in organ procurement.
- CarpeDiem (SCRIPT Dataset v1.8.0)^17^ is an ICU research resource containing per-day clinical data from 690 mechanically ventilated patients suspected of pneumonia, totaling 15 287 ICU-days. It includes demographics, pneumonia adjudication labels, outcomes, daily vital signs, laboratory results, and mechanical support parameters, enabling fine-grained temporal analysis of critical illness trajectories.
- INSPIRE ^18^ is a perioperative dataset comprising approximately 130 000 surgical cases collected between 2011 and 2020 at a South Korean academic hospital. It includes patient demographics, diagnoses, procedural codes, anesthesia types, high-resolution intraoperative vital signs, longitudinal laboratory results, medication administration data, and outcomes such as length of stay and mortality.

##### Ethical approval

The research was determined to be exempt from IRB approval by the MIT Committee on the Use of Humans as Experimental Subjects (E-7730).

#### LLM-as-a-judge evaluation

We implemented a blinded comparative evaluation using *GPT-4o* as an independent judge. For each variable, the judge was presented with a randomized set of candidate descriptions, including the official documentation and outputs generated by *GPT-4*, *GPT-4o-mini*, *Gemini-2.5-Flash*, and *Llama 3.1*.

The judge received structured contextual information (domain, column name, data type, and sample values) but was blinded to the source of each description. For each case, the judge selected the most accurate description and provided a qualitative justification based on clarity, semantic correctness, and consistency with the metadata.

This procedure enabled the computation of comparative win rates across models and against expert-curated documentation, identifying scenarios in which automated descriptions matched or exceeded existing reference standards.

#### Human expert evaluation

A structured expert evaluation was conducted on the datasets described in section “Evaluation datasets.” For each column, a domain expert was presented with the automatically generated description and the corresponding official documentation in anonymized form to prevent source bias. Descriptions were rated along 2 dimensions:

1. **Correctness (0–5)**: degree to which the description accurately captures the clinical meaning and functional role of the variable.
2. **Hallucination (0–2)**: presence of unsupported, inferred, or fabricated clinical details not grounded in the provided metadata.

#### Practical utility of generated dictionaries

To assess the practical utility of the generated data dictionaries, we evaluated their use across 2 downstream settings: documentation auditing and augmentation of a text-to-SQL task.

**Documentation auditing task.** To assess the quality of dataset documentation, we formulated a documentation auditing task in which an LLM-based *Senior Auditor* agent evaluates both official and generated descriptions. For each dataset column, the auditor is provided with (1) the column name, (2) its data type, (3) a set of sample values extracted from the dataset, (4) the official documentation, and (5) the description generated by DataAtlas. The auditor is prompted to cross-check these sources and identify documentation issues. Specifically, we evaluate 4 failure modes: (1) **Inaccuracy**, defined as contradictions between the official description and the actual data type or sample values; (2) **Ambiguity**, arising from the use of undefined technical acronyms or vague terminology; and (3) **Resolution**, assessing whether the generated description addresses the identified issues.

For each column, the auditor produces a structured output including: the presence and type of documentation error, its severity, a textual justification grounded in the data, and a binary judgment indicating whether the generated description improves upon the official one. Additionally, the auditor proposes a revised “gold-standard” description that integrates the most accurate and complete information from both sources. This procedure enables a data-driven comparison between official and generated documentation, allowing us to quantify the prevalence of documentation issues and the ability of DataAtlas to resolve them.

**Text-to-SQL task.** To evaluate the practical utility of the generated documentation, we formulated documentation quality as a text-to-SQL task. We manually curated a gold-standard SQL benchmark consisting of 25 clinically meaningful natural language questions covering key aspects of the CarpeDiem dataset, including demographics, clinical outcomes, procedures, laboratory measurements, and severity scores. Each question was paired with a manually verified SQL query whose execution was validated against the raw dataset to ensure correctness. We evaluated 2 experimental conditions: (1) *Schema-only*, where the LLM is provided with the database schema alone, and (2) *Schema+Dictionary*, where the schema is augmented with the automatically generated data dictionary. For each question, the model is prompted to generate a SQL query, which is then executed against the dataset and compared to the gold-standard query output. To account for the stochastic nature of LLM generation, the entire benchmark was repeated 15 times for each condition. This allowed us to estimate the stability of the results and compute summary statistics across runs. We report *Execution Accuracy vs Gold* (EA${\;}_{\text{gold}}$), defined as the fraction of generated queries whose execution results exactly match the output of the corresponding gold-standard SQL query. For each condition, we report the mean EA${\;}_{\text{gold}}$, the standard deviation across runs, and the corresponding 95% confidence interval. The complete set of benchmark questions and gold SQL queries is provided in the Supplementary Material.

## Results

We evaluated DataAtlas along 3 dimensions: the quality of the generated semantic descriptions, the accuracy of inferred relational structures, and the practical utility of the resulting data dictionaries on downstream tasks. When applied across 3 heterogeneous clinical datasets, DataAtlas produced descriptions that were frequently preferred to official documentation, showed strong agreement with expert review, and improved performance in a text-to-SQL benchmark.

### LLM-as-a-judge evaluation

We conducted a blinded pairwise evaluation using *GPT-4o* as an independent judge to compare the reference (“official”) dataset documentation against descriptions generated by 4 model architectures (*GPT-4*, *GPT-4o-mini*, *Gemini-2.5-Flash*, and *Llama 3.1*). For each column, the judge was presented with anonymized descriptions in randomized order and asked to select the one that provided greatest clarity and clinical completeness. Win rates were computed over all evaluated columns.

#### Comparative win rates

Table 2 reports the percentage of pairwise comparisons in which the official documentation was preferred. Lower win rates for the reference source indicate higher relative quality of the generated descriptions.

**Table 2.** The results are presented as the percentage of times that the official description was preferred to the generated one for each column in the datasets.

Comparison	ORCHID (*n*=64)	CarpeDiem (*n*=101)	INSPIRE (*n*=62)
**Official vs GPT-4**	53.1%	39.6%	4.8%
**Official vs GPT-4o-mini**	39.1%	43.6%	32.3%
**Official vs Gemini-2.5-Flash**	53.1%	40.6%	33.9%
**Official vs Llama 3.1**	71.9%	61.4%	19.4%

Lower values indicate a stronger relative performance of the generated descriptions. For example, for ORCHID, among all the 64 columns evaluated, *GPT-4* preferred the official description 53.1% of the time.

Across datasets, automated descriptions were frequently preferred to official documentation. The strongest performance was observed for the INSPIRE dataset, where *GPT-4* descriptions were preferred in 95.2% of comparisons (official win rate: 4.8%). Conversely, *Llama 3.1* demonstrated the weakest performance, with official documentation preferred in 71.9% of cases for ORCHID and 61.4% of cases for CarpeDiem.

#### Qualitative patterns

Qualitative review of the judge justifications indicated that (1) the tool expands minimal descriptions into meaningful explanations, as shown in the representative comparisons in Table 3 (eg, the official description for “ethnicity” is simply “ethnicity,” while the generated description is “Ethnicity of patients, categorized as Hispanic, Latino, Not Hispanic or Latino, or Unknown/Not Reported”); (2) the tool is also able to accurately expand acronyms (eg, the official description for opo (“Anonymized OPO ID”) was consistently ranked below the *GPT-4* description (“Categorical identifier for the Organ Procurement Organization involved in organ retrieval”)). The model (3) explicitly states the data type of the values in the columns, which the official documentation often omits. In some cases, (4) the tool demonstrates its ability to provide medical interpretations rather than just structural descriptions (eg, “PaO_2_/FiO_2_” in the official documentation versus “PaO_2_/FIO_2_ ratio measures the ratio of arterial oxygen partial pressure to fractional inspired oxygen” in the generated one).

#### Structural integrity and pattern recognition

The pattern-masking component captures recurring structural formats in column values (eg, mapping “OPO3_P796373” to “AAA9_A999999”), exposing latent regularities that are not explicitly documented. This abstraction supports the interpretation of otherwise opaque values (eg, composite identifiers, temporal encodings, and categorical codes), and provides useful information for downstream semantic type inference. In turn, these pattern-aware representations contribute to the generation of more precise and contextually grounded descriptions.

### Human expert evaluation

**Figure 2.**
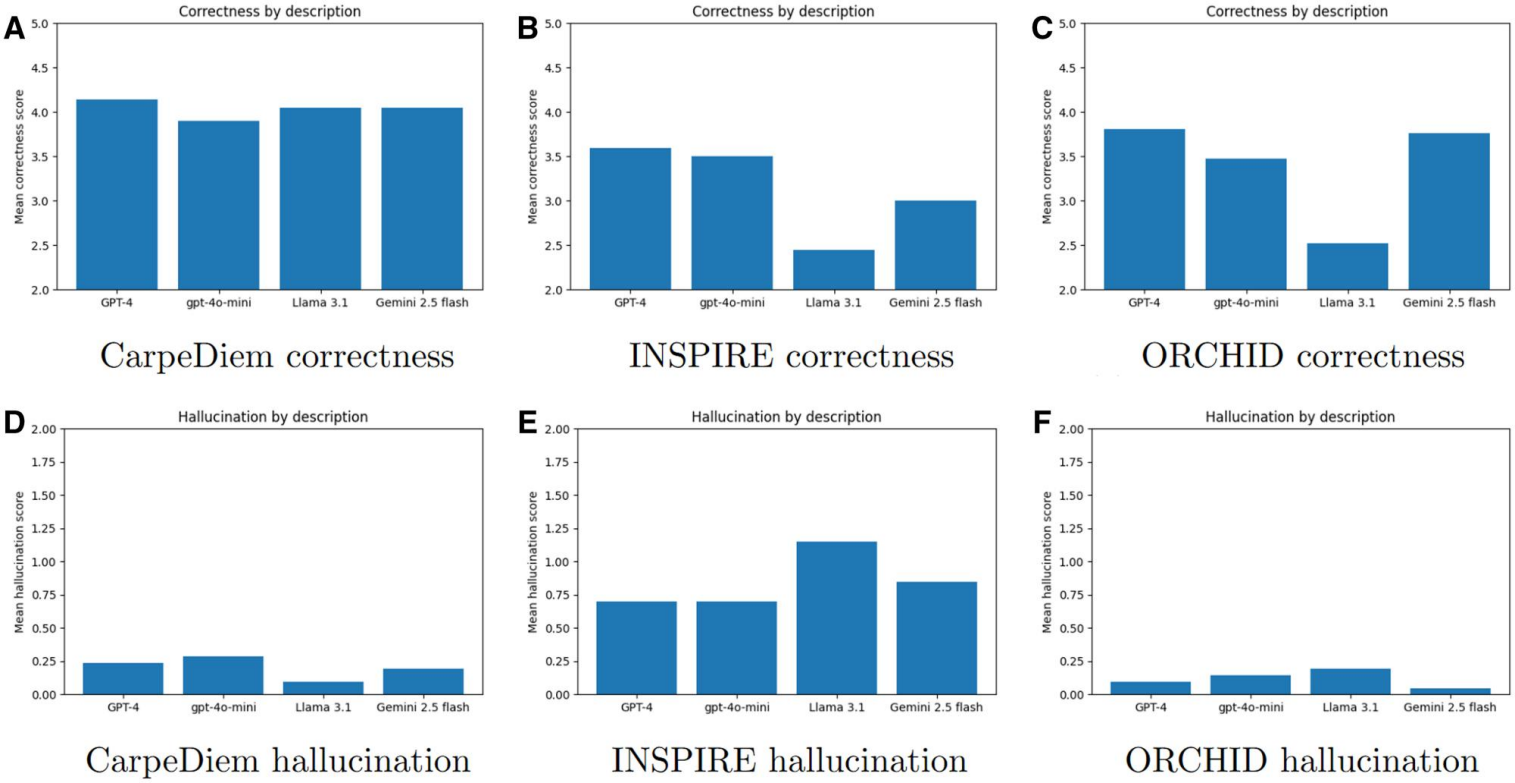
Human evaluation results across 4 model architectures. Top row: correctness scores. Bottom row: hallucination scores.

To assess the clinical reliability and semantic precision, we conducted a domain-expert review focusing on the datasets discussed in section “Evaluation datasets.” For each dataset, a domain expert evaluated 21 representative columns across 4 model architectures: *GPT-4* (Description A), *GPT-4o-mini* (Description B), *Llama 3.1* (Description C), and *Gemini-2.5-Flash* (Description D). Results are shown in Figure 2.

#### Correctness

The expert review results broadly corroborated the LLM-as-a-judge findings. *GPT-4* and *GPT-4o-mini* were identified by the expert as the most reliable models, frequently achieving *fully correct* (5/5) ratings. Expert feedback suggested that these models provided the most informative descriptions, particularly when representative values allowed the model to make implicit coding schemes explicit (eg, binary mappings such as 1=yes, 0=no).

A recurring challenge across all architectures was the distinction between ICU-specific and general hospital-level events. In several cases, models inferred ICU admission timestamps rather than broader hospital admission timestamps, contributing to “partially correct” ratings.

#### Hallucination

Most generated descriptions were rated as having no hallucination. When hallucinations occurred, they generally reflected over-interpretation of clinical context rather than arbitrary fabrication. Three main failure modes were observed: contextual misattribution (eg, assuming an ICU setting for general hospitalization data), physiological misinterpretation (eg, incorrectly inferring temperature units), and confusion between related clinical measurements (eg, SpO_2_ versus SaO_2_).

Overall, the expert review indicated that the “contextual anchoring” provided by representative sample values is critical for grounding model outputs. Without this anchoring, models were more likely to default to generic interpretations and produce technically plausible but imprecise descriptions.

### Practical utility of generated data dictionaries

#### Document auditing and error discovery

The auditing framework revealed some documentation issues across all evaluated datasets (CarpeDiem, ORCHID, and INSPIRE), as illustrated in Table 4. The most common failure modes were **inaccuracy** and **ambiguity**, often arising from mismatches between official descriptions and the underlying data or from the use of undefined domain-specific terminology.

**Table 3.** Representative comparison of official vs *GPT-4* generated descriptions.

Column	Official	GPT-4	Preferred	Justification
**OPOReferrals.opo**	Anonymized OPO ID	Categorical identifier for the Organ Procurement Organization involved in organ retrieval.	GPT-4	Description 2 is more complete and aligns with the context by specifying that the identifier is for the Organ Procurement Organization involved in organ retrieval, which is relevant to the domain of organ donation.
**OPOReferrals.gender**	Patient sex/gender	Column gender represents the sex of the organ retrieval patient, denoted as F or M.	GPT-4	Description 2 is more complete and specific, clearly indicating that the column represents the sex of the organ retrieval patient and explaining the meaning of the values “F” and “M.”
**OPOReferrals.race**	Patient race	Column race indicates the ethnicity of the organ retrieval patient.	Official	Description 1 is more accurate as it correctly identifies the column as representing “race” rather than “ethnicity,” which aligns with the sample values provided.
**OPOReferrals.tissue_referral**	Was patient also referred for tissue donation?	Indicates whether tissue was referred for organ retrieval, represented as a binary flag.	Official	Description 1 is clearer and more accurately reflects the context of a referral for tissue donation, aligning with the Boolean data type and sample values. Description 2 incorrectly suggests a focus on organ retrieval rather than tissue donation.

The table highlights the tradeoffs between semantic richness and technical precision observed during evaluation.

**Table 4.** Examples of documentation issues identified by the proposed auditing framework across the 3 evaluated datasets.

Column	Official_desc	Generated_desc	Error type	Findings
**Dataset: *CarpeDiem***				
**Patient/ICU stay/ICU day**	(missing)	Timestamp marking a patient’s ICU stay and the day within that stay.	Inaccuracy	The official documentation is missing despite the column encoding structured information combining patient ID, ICU stay, and ICU day.
**External_transfer_flag**	0/1 flag set if the patient was transferred from an external facility for this hospitalization.	Binary indicator showing whether a patient was transferred from an external facility.	Inaccuracy	The wording introduces ambiguity despite the column representing a simple binary indicator.
**COVID_status**	True/false flag of whether patient had COVID.	Binary floating-point indicator (1.0 positive, 0.0 negative).	Inaccuracy	The documentation implies a Boolean variable while the stored values are floating-point.
**Dataset: *ORCHID***				
**opo**	Anonymized OPO ID	Categorical identifier for the Organ Procurement Organization involved in organ retrieval.	Ambiguity	The official description uses the acronym “OPO” without definition, creating ambiguity for new users.
**gender**	Patient sex/gender	Biological sex of the patient, represented as F or M.	Inaccuracy	The official description conflates sex and gender while the values correspond to binary biological sex categories.
**tissue_referral**	Was patient also referred for tissue donation?	Indicates whether the patient was considered for tissue donation (true/false).	Inaccuracy	The original description is inconsistent with the Boolean data type and sample values.
**Dataset: *INSPIRE***				
**abbreviations**	(missing)	Abbreviations for healthcare departments used in the dataset.	Inaccuracy	The official documentation is missing despite the column containing structured department abbreviations.
**chart_time**	The time for the diagnosis recorded	Time recorded in elapsed minutes from a reference point.	Inaccuracy	Values are integers including negative numbers, indicating elapsed time rather than a timestamp.
**item_name**	Label for the measurement	Name of the laboratory test (eg, glucose, creatinine).	Ambiguity	The official description is too generic and does not convey that the column contains specific laboratory test names.

In the CarpeDiem dataset, several discrepancies were identified between documentation and actual data representation. For instance, the composite key Patient/ICU stay/ICU day lacked any official description, yet DataAtlas successfully reconstructed its structured semantic meaning. Similarly, the COVID_status variable was described as Boolean, while the observed values were stored as floating-point indicators (0.0/1.0), highlighting a clear inconsistency.

Across the ORCHID dataset, ambiguity emerged as a dominant issue, particularly due to the use of undefined acronyms (eg, OPO) and unclear identifier structures. The generated descriptions resolved these ambiguities by expanding acronyms and providing explicit semantic context. In addition, inconsistencies in terminology, such as the interchangeable use of “sex” and “gender,” were identified and corrected.

In the INSPIRE dataset, the audit uncovered missing or generic documentation. Several columns (eg, department abbreviations) lacked any official description, while others (eg, chart_time) were inaccurately described as timestamps despite representing elapsed time encoded as integers, including negative values. The generated descriptions improved clarity by grounding explanations in the observed data distribution.

Overall, these results demonstrate that DataAtlas not only identifies documentation errors but also improves semantic clarity by aligning descriptions with actual data characteristics.

#### Executable text-to-SQL task

**Figure 3.**
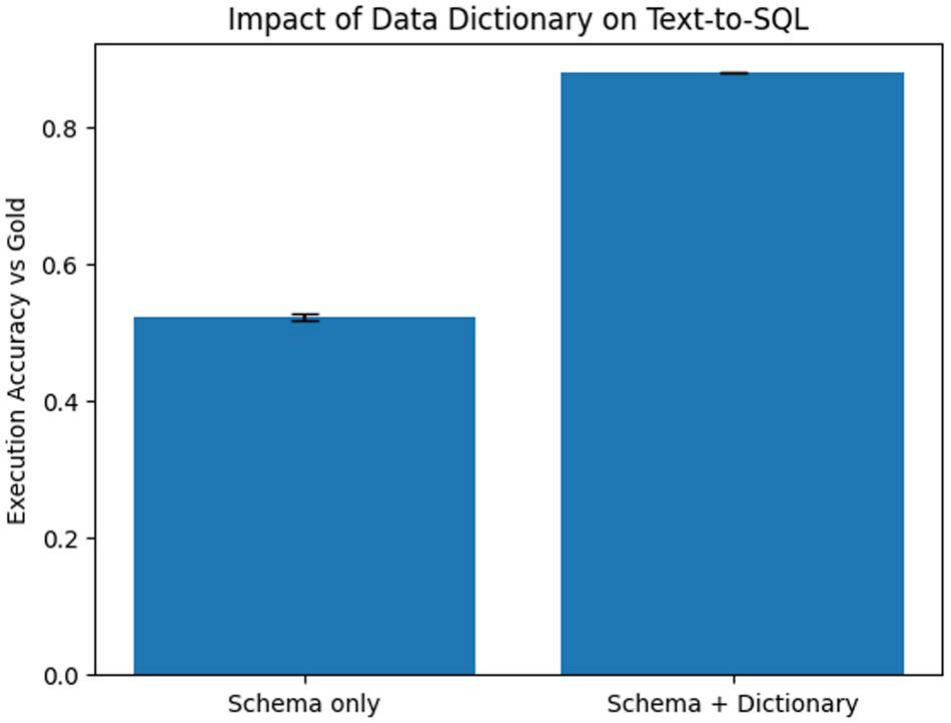
Impact of automatically generated data dictionaries on text-to-SQL performance in the CarpeDiem dataset. Execution accuracy (EA${\;}_{\text{gold}}$) increases from 0.52±0.01 to 0.88±0.00 (+35.7%) when the schema is augmented with the generated dictionary, highlighting the role of high-quality semantic documentation in enabling correct query generation.

Augmenting the schema with the generated data dictionary resulted in a substantial improvement in query generation accuracy. Exact-match accuracy EA${\;}_{\text{gold}}$ increased from **0.523±0.01** to **0.880±0.00**, where the reported ± values represent the standard deviation across 10 independent runs (95% confidence interval), corresponding to a gain of 35.7%. TAs illustrated in Figure 3, the addition of semantic documentation not only improved overall performance but also increased the stability of the model’s outputs across repeated runs. While schema-only prompting showed small variability in accuracy, the schema augmented with the generated data dictionary produced consistent results across all runs, indicating that the additional semantic context helps the model reliably interpret column meanings and generate correct SQL queries. These findings highlight the importance of rich semantic metadata for enabling LLMs to interact effectively with complex clinical datasets.

## Discussion

In this study, DataAtlas generated structured data dictionaries for complex tabular datasets by combining deterministic structural profiling with LLM-based semantic inference. Across multiple evaluation settings, the approach improved the interpretability of raw clinical data, and the generated descriptions were frequently preferred to existing documentation. These findings suggest that LLM-assisted documentation may help address a persistent barrier to secondary data use: the gap between shared data files and the structured metadata needed to interpret them.

Generated descriptions were frequently preferred to official, human-curated documentation, particularly when existing descriptions were brief, ambiguous, or included acronyms. In these cases, LLM-generated descriptions incorporated contextual signals from the data to produce more complete explanations. This does not suggest that automated descriptions should replace curated documentation. Instead, it suggests that LLM-assisted tools can augment documentation workflows, identify omissions, and improve the usability of shared datasets. From a practical standpoint, this hybrid approach shifts the annotation workflow from time-intensive manual curation to rapid automated generation followed by targeted expert review, substantially reducing overall human effort.

Human evaluation highlighted an important boundary of automated generation of descriptive text. Although the best-performing models often generated useful descriptions, some models introduced subtle misinterpretations that would be difficult to detect through automated evaluation alone. Errors included, for example, incorrect inference of units and confusion between closely related physiological measurements. These findings underline the importance of a continued role for human review, particularly in cases where semantic errors may have significant downstream consequences.

The practical impact of semantic enrichment offered by DataAtlas is visible in the text-to-SQL benchmark. Augmenting a schema with a generated dictionary improved the execution accuracy of queries. This suggests that the value of automated metadata generation is not limited to human readability; the resulting documentation may also improve machine-mediated data access by helping downstream systems disambiguate variable meaning and table structure.

DataAtlas directly supports FAIR (Findable, Accessible, Interoperable, Reusable)^19^ data principles by automating the generation of structured, machine-readable metadata. The generated data dictionaries enhance Findability by providing searchable descriptions of dataset content, improve accessibility by clarifying variable meanings and data structures, facilitate interoperability by documenting data types, coding schemes, and relational structures in standardized formats (JSON, SQL), and promote reusability by reducing barriers to dataset interpretation for secondary analyses. For data repositories and research consortia, automated dictionary generation can help scale FAIR compliance across large collections of heterogeneous datasets.

Despite the clinical utility demonstrated by DataAtlas, several limitations remain that warrant consideration for future research and practical deployment. First, the comparative evaluation revealed a tradeoff between semantic richness and technical precision. LLM-based descriptions often provide richer context, explicitly expanding abbreviations or linking variables to broader clinical processes (eg, OPOReferrals.opo in Table 3). However, deterministic, official descriptions occasionally better capture strictly structural metadata roles, such as primary or foreign key designations (eg, CBCEvents.row_id and CultureEvents.row_id). This highlights that while LLMs excel at natural language explanations, authoritative sources remain valuable for accurately documenting structural database properties.

Second, the system is vulnerable to domain hallucination. As seen in the evaluation of OPOReferrals.tissue_referral and OPOReferrals.race (see Table 3), the model occasionally misinterpreted the specific clinical intent, for example, confusing “race” with “ethnicity” or misrepresenting the purpose of a referral flag. These instances, though infrequent, highlight the risk of “domain hallucination,” where the model’s prior knowledge may override the context provided by sample values.

Third, performance depended on the quality and diversity of the provided sample values. Sparse or non-representative samples may lead the LLM to generate descriptions that are technically correct but clinically misleading or overly generic. While the DataAtlas successfully expanded common acronyms like SNF or LTACH, less frequent or project-specific codes that are absent from the schema may not be fully captured.

Finally, model choice affected performance. Although DataAtlas was able to run on consumer-grade hardware, smaller locally deployed models such as *Llama 3.1* showed reduced descriptive quality relative to larger proprietary models. This creates a practical tradeoff between privacy, cost, and performance. For institutions operating in restricted environments, local deployment may still be attractive, but the reduction in model capacity may limit semantic precision.

Taken together, these findings suggest that DataAtlas is best viewed as an assistive system for documentation and dataset understanding rather than a replacement for expert curation. Potential use cases include early-stage dataset familiarization, auditing of existing data dictionaries, support for repositories hosting heterogeneous tabular data, and augmentation of downstream analytical workflows. Future work should focus on improving robustness to local coding conventions, strengthening safeguards against domain hallucination, and evaluating the approach in non-clinical settings.

### Data privacy

DataAtlas does not store, retain, or share any input data. When deployed with local models (eg, *Llama 3.1* via Ollama), all processing occurs on the local machine with zero external data transmission. When using cloud-based API models (*GPT* and *Gemini*), only the extracted metadata (column names, data types, statistical summaries, and sample values) are transmitted to the respective API endpoints, subject to those providers’ data handling policies. For sensitive or regulated clinical data, we strongly recommend local deployment with open-weights models to maintain complete data sovereignty and comply with institutional data governance requirements. This approach ensures that no patient-level information leaves the secure computing environment.

## Conclusion

DataAtlas is an automated pipeline for generating structured data dictionaries from tabular datasets using deterministic profiling and LLM-based semantic inference. The system is able to generate accurate descriptions, identify inconsistencies in official records, and infer relationships among tables.

These findings suggest that automated data dictionary generation may improve both human and machine use of shared datasets. In addition to supporting dataset interpretation and auditing, the generated metadata improved text-to-SQL performance, highlighting the broader value of semantic documentation for computational data access.

Although limitations remain, particularly with respect to hallucination risk, sample quality, and model selection, DataAtlas implements a practical approach to reducing the documentation burden associated with complex tabular data. By helping transform poorly documented tables into more interpretable and reusable resources, it may strengthen the reproducibility and utility of shared scientific datasets.

## Supplementary Material

ooag119_Supplementary_Data

## Data Availability

The datasets analyzed in this study are publicly available. All the datasets used in this study are available through PhysioNet upon completion of the required credentialing process and data use agreements. Additional materials generated during the current study are available from the corresponding author upon reasonable request.
