# Supplementary material for: DataAtlas: automatic generation of data dictionaries using large language models
Source: JAMIA Open. 2026 Jun 27;9(3):ooag119. doi: 10.1093/jamiaopen/ooag119 (PMC13310032; doi:10.1093/jamiaopen/ooag119)
Supplement: ooag119_Supplementary_Data [file ooag119_supplementary_data.zip › supplementary.pdf]

Supplementary Material: Text-to-SQL Benchmark Queries

Appendix A: Text-to-SQL Benchmark Queries

The benchmark consists of 25 clinically meaningful natural language questions paired with manually verified gold-standard SQL queries executed on the `CarpeDiem` dataset.

Table 1: Natural language questions and corresponding gold SQL queries used in the Text-to-SQL benchmark.

**Question**	**Gold SQL Query**
How many distinct patients are recorded in the dataset?	`SELECT COUNT(DISTINCT Patient_id) FROM carpediem_v1_8_dataset`
What is the average age of patients in the dataset?	`SELECT AVG(Age) FROM carpediem_v1_8_dataset`
How many patients had a bad outcome during hospitalization?	`SELECT COUNT(DISTINCT Patient_id) FROM carpediem_v1_8_dataset WHERE Binary_outcome = 1`
Count the number of patients whose discharge disposition was 'Died'.	`SELECT COUNT(DISTINCT Patient_id) FROM carpediem_v1_8_dataset WHERE Discharge_disposition = 'Died'`
What is the average BMI of all patients?	`SELECT AVG(BMI) FROM carpediem_v1_8_dataset`
What is the maximum SOFA score recorded across all ICU days?	`SELECT MAX(SOFA_score) FROM carpediem_v1_8_dataset`
What is the average cumulative ICU days grouped by gender?	`SELECT Gender, AVG(Cumulative_ICU_days) FROM carpediem_v1_8_dataset GROUP BY Gender`
How many patients were COVID positive?	`SELECT COUNT(DISTINCT Patient_id) FROM carpediem_v1_8_dataset WHERE COVID_status = 1`

**Question**	**Gold SQL Query**
How many patients were immunocompromised?	`SELECT COUNT(DISTINCT Patient_id) FROM carpediem_v1_8_dataset WHERE Immunocompromised_flag = 1`
How many patients received ECMO during their ICU stay?	`SELECT COUNT(DISTINCT Patient_id) FROM carpediem_v1_8_dataset WHERE ECMO_flag = 1`
How many patients received both CRRT and Hemodialysis during hospitalization?	`SELECT COUNT(DISTINCT Patient_id) FROM carpediem_v1_8_dataset WHERE CRRT_flag = 1 AND Hemodialysis_flag = 1`
What is the average heart rate across all ICU records?	`SELECT AVG(Heart_rate) FROM carpediem_v1_8_dataset`
What is the lowest systolic blood pressure recorded?	`SELECT MIN(Systolic_blood_pressure) FROM carpediem_v1_8_dataset`
What is the maximum lactic acid level recorded in the dataset?	`SELECT MAX(Lactic_acid) FROM carpediem_v1_8_dataset`
What is the average diastolic blood pressure for patients older than 70?	`SELECT AVG(Diastolic_blood_pressure) FROM carpediem_v1_8_dataset WHERE Age > 70`
What patients received Remdesivir during hospitalization?	`SELECT COUNT(DISTINCT Patient_id) FROM carpediem_v1_8_dataset WHERE Remdesivir_received = 1`
What patients received Tocilizumab during hospitalization?	`SELECT COUNT(DISTINCT Patient_id) FROM carpediem_v1_8_dataset WHERE Tocilizumab_received = 1`
What patients received both Remdesivir and Tocilizumab?	`SELECT COUNT(DISTINCT Patient_id) FROM carpediem_v1_8_dataset WHERE Remdesivir_received = 1 AND Tocilizumab_received = 1`
How many patients had bronchoalveolar lavage (BAL) performed?	`SELECT COUNT(DISTINCT Patient_id) FROM carpediem_v1_8_dataset WHERE BAL_performed = 1`
How many patients had pathogen resistance markers detected on BAL?	`SELECT COUNT(DISTINCT Patient_id) FROM carpediem_v1_8_dataset WHERE Pathogen_resistance_detected = 1`
How many pneumonia episodes were bacterial or viral and cured?	`SELECT COUNT(DISTINCT Episode_id) FROM carpediem_v1_8_dataset WHERE Episode_etiology = 'Bacterial/viral' AND Episode_is_cured = 'Cured'`

Question	Gold SQL Query
How many pneumonia episodes lasted longer than 7 days?	`SELECT COUNT(DISTINCT Episode_id) FROM carpediem_v1_8_dataset WHERE Episode_duration > 7`
What is the average SOFA score per ICU stay?	`SELECT Patient_id, ICU_stay, AVG(SOFA_score) FROM carpediem_v1_8_dataset GROUP BY Patient_id, ICU_stay`
What is the average lactic acid level for patients with multiple ICU stays?	`SELECT Patient_id, AVG(Lactic_acid) FROM carpediem_v1_8_dataset WHERE Number_of_ICU_stays > 1 GROUP BY Patient_id`
How many patients had virus clearance within 5 ICU days of the episode?	`SELECT COUNT(DISTINCT Patient_id) FROM carpediem_v1_8_dataset WHERE Virus_cleared_on IS NOT NULL AND Virus_cleared_on <= 5`
