# Supplementary material for: DataAtlas: automatic generation of data dictionaries using large language models
Source: JAMIA Open. 2026 Jun 27;9(3):ooag119. doi: 10.1093/jamiaopen/ooag119 (PMC13310032; doi:10.1093/jamiaopen/ooag119)
Supplement: ooag119_Supplementary_Data [file ooag119_supplementary_data.zip › appendix_B_dataatlas.pdf]

Supplementary Material: Clinical Relationship Discovery

Appendix B: Schema Reconstruction with DataAtlas

This appendix describes the schema reconstruction module implemented in `DataAtlas`. Unlike traditional database reverse-engineering approaches that rely exclusively on explicit foreign key constraints, the proposed pipeline reconstructs a relational schema from raw dataset metadata using a combination of statistical profiling and large language model (LLM) inference.

`DataAtlas` operates in three stages: (i) metadata summarization, (ii) LLM-based schema inference, (iii) deterministic validation and SQL generation.

Metadata Representation

For each table and column, the system constructs a compact representation including:

- inferred data types;
- uniqueness and null-value ratios;
- candidate key detection signals;
- sample values;
- LLM-generated descriptions.

A column is flagged as a *candidate key* when it satisfies: (i) high uniqueness ratio (≥ 0.98), (ii) low missingness (≤ 0.01), and (iii) presence of identifier-like keywords (e.g., `id`, `uuid`, `key`).

LLM-Based Schema Reconstruction

The summarized metadata is provided to an LLM with strict constraints requiring it to:

- identify primary keys;
- infer foreign key relationships;
- assign relationship cardinalities.

The model is explicitly constrained to use only observed metadata and is instructed to avoid hallucinated tables, columns, or relationships.

The output is required to follow a strict JSON schema:

```
{
  "tables": [
    {
      "table_name": "...",
      "primary_keys": ["..."],
      "foreign_keys": [
        {
          "from_table": "...",
          "from_column": "...",
          "to_table": "...",
          "to_column": "...",
          "cardinality": "many-to-one"
        }
      ]
    }
  ]
}
```

Example Output Schema

Figure 1 shows a DBML representation of a reconstructed subset of the `MIMIC-IV` dataset, generated by importing the reconstructed PostgreSQL schema into dbdiagram.io.

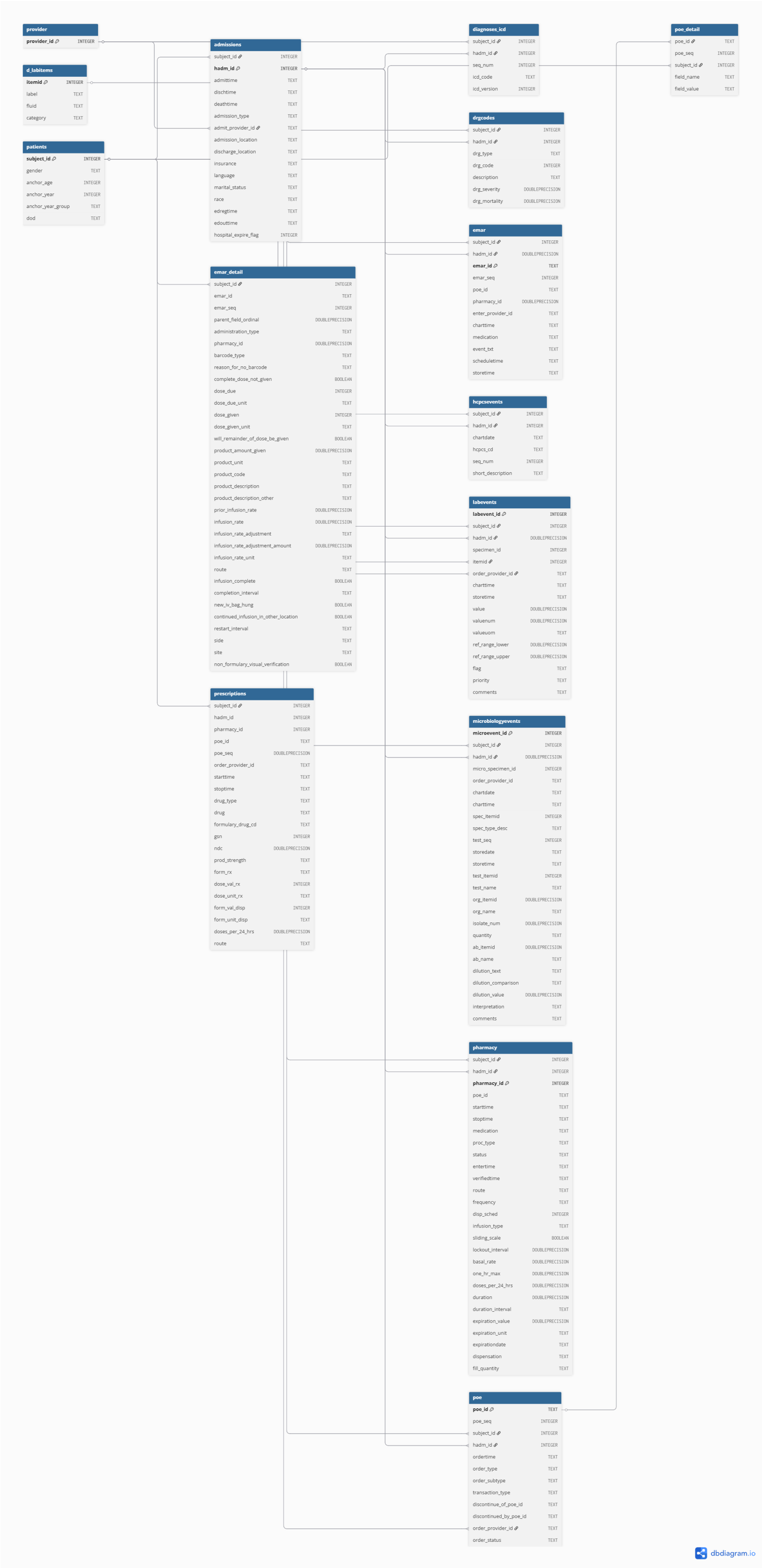

Figure 1: Example relational schema reconstructed by `DataAtlas` on a subset of the `MIMIC-IV` dataset. Nodes represent tables, while inferred foreign keys define inter-table relationships.

Key Observations

On the evaluated subset of `MIMIC-IV`, the system successfully reconstructs core clinical relational structure, including:

- patient-to-admission linkage via `subject_id`;
- admission-centric clinical event aggregation via `hadm_id`;
- medication and prescription tracking via `poe_id` and `pharmacy_id`;
- laboratory and microbiology event alignment via provider and order identifiers.

These reconstructed relationships reflect both explicit administrative keys and implicit operational dependencies across clinical workflows.

Deterministic SQL Generation

The inferred schema is transformed into PostgreSQL DDL using a deterministic mapping layer.

Data types are normalized as follows:

- `integer` → `INTEGER`
- `float/double` → `DOUBLE PRECISION`
- `boolean` → `BOOLEAN`
- `timestamp/datetime` → `TIMESTAMP`
- fallback → `TEXT`

Primary keys and foreign keys are explicitly encoded into SQL constraints to ensure direct deployability in relational database systems.

Summary

Overall, `DataAtlas` provides a hybrid pipeline that combines extracted metadata and LLM reasoning to reconstruct relational database schemas from heterogeneous clinical datasets without relying on pre-existing foreign key definitions.
